# Navigating STEM careers with AI mentors: a new IDP journey

**DOI:** 10.3389/frai.2024.1461137

**Published:** 2024-10-08

**Authors:** Chi-Ning Chang, John Hui, Cammie Justus-Smith, Tzu-Wei Wang

**Affiliations:** School of Education, Virginia Commonwealth University, Richmond, VA, United States

**Keywords:** individual development plan, myIDP, career development, career planning, STEM mentoring, human-AI mentoring, mentorship, large language model

## Abstract

**Introduction:**

Mentoring is crucial to the success of STEM higher education. The Individual Development Plan (IDP) is a common career development tool in STEM graduate education that facilitates structured mentor-mentee interactions and goal setting. This study examined the integration of AI mentors into the myIDP framework to provide real-time support and career insights.

**Methods:**

Using Google Gemini as an AI mentor, this study developed and assessed AI prompts within the myIDP framework. Eighteen STEM graduate students, primarily from underrepresented groups, were trained to engage with the AI mentor. Their interactions, feedback, and comments were analyzed using sentiment and thematic analysis.

**Results:**

Participants reported positive experiences with AI mentors, noting benefits, such as immediate responses, up-to-date information, access to multiple AI mentors, enhanced ownership of career development, and time savings. However, concerns about misinformation, bias, privacy, equity, and algorithmic influences have also been raised. The study identified two hybrid human-AI mentoring models—Sequential Integration and Concurrent Collaboration—that combine the unique strengths of human and AI mentors to enhance the mentoring process.

**Discussion:**

This study underscores the potential of AI mentors to enhance IDP practices by providing timely feedback and career information, thereby empowering students in their STEM career development. The proposed human-AI mentoring models show promise in supporting underrepresented minorities, potentially broadening participation in STEM fields.

## 1 Introduction

Mentoring plays a critical role in advancing success in higher education (U.S. Department of Education, 2012). With the growing emphasis on career readiness and global competitiveness in the fields of science, technology, engineering, and mathematics (STEM) (National Science Foundation, 2022), career development has become a fundamental component of STEM mentoring. This form of mentoring goes beyond basic guidance, establishing a strategic framework for navigating educational and professional challenges. By accelerating skill development, offering industry insights, fostering networking, and providing essential career and psychosocial support, effective mentorship is crucial for thriving in the competitive STEM landscape (National Academies of Sciences, Engineering, and Medicine, 2020).

Recognizing the value of mentorship, the U.S. CHIPS and Science Act of 2022, along with various federal funding agencies [e.g., National Science Foundation (NSF)], has emphasized the integration of mentorship into workforce development, particularly in sectors like semiconductor manufacturing, where aligning academic preparation with industry needs is critical. The act mandates that all NSF-supported graduate students utilize Individual Development Plans (IDPs), a widely recognized tool in STEM graduate education, to map educational goals, facilitate career exploration, and guide professional development in collaboration with principal investigators or mentors (CHIPS and Science Act, 2022; National Science Foundation, 2024). By fostering structured, two-way communication within the mentor-mentee relationship, IDPs help students achieve their career aspirations (Chang et al., 2021; Hobin et al., 2012), thereby supporting the development of a diverse and skilled workforce essential for maintaining U.S. leadership in global science and technology.

myIDP is a commonly used web-based IDP platform^1^ developed in 2012 by the American Association for the Advancement of Science (AAAS), the Federation of American Societies for Experimental Biology (FASEB), and experts from multiple universities (Fuhrmann et al., n.d.). It has gained significant popularity in U.S. universities, enabling graduate students to self-assess, explore different career paths, and set SMART (Specific, Measurable, Achievable, Relevant, and Time-Bound) goals with the assistance of mentoring teams. During the self-assessment stage, myIDP offers assessments for scientific skills, career interests, and work-related values. Based on the assessment results, the platform provides users with a variety of career options, each with a matching score to help them consider career fit. Additionally, myIDP offers resources including articles, books, and professional societies to help users understand each career path. To further explore their target career, myIDP provides tips for attending relevant events and networking with professionals. Once a career path is selected, users are prompted to create SMART goals for career advancement, skill improvement, and project development. The platform also offers tips on identifying a mentoring team to discuss these goals and support the career development process. Upon completion, a certificate can be generated, which is often part of the paperwork required to meet degree or program requirements at some universities (Chang et al., 2023).

Due to the rapid evolution of STEM professions over the last decade, some resources about current career options on myIDP are limited and outdated. For example, as of July 1, 2024, the suggested books for each career path were mostly published before 2010, with publication dates ranging from 1993 to 2015. Consequently, student users working with myIDP rely heavily on human mentors for support. The drawback is that mentees cannot receive immediate feedback and up-to-date career information from human mentors due to time limitations and knowledge blind spots. In light of the advancements and applications of Large Language Models (LLMs), such as those used by Google Gemini and ChatGPT, this study explores the feasibility of using artificial intelligence (AI) mentors to enhance myIDP practice. We hypothesize that graduate students can receive real-time support throughout the process from AI mentors, while acknowledging that this integration might also face challenges (Köbis and Mehner, 2021).

Recent advancements in Generative Pre-trained Transformer (GPT) models have significantly influenced various industries around the world (Dehouche, 2021; ChatGPT Generative Pre-trained Transformer and Zhavoronkov, 2022; Li, 2020). In educational research, notable examples of these applications are the language processing AI systems ChatGPT and Gemini. These systems are built on Large Language Models (LLMs), which are so-called because of the substantial memory required for their training, maintenance, and optimization. LLMs operate using algorithms that analyze extensive text data to identify patterns and relationships within text. Through this training, LLMs develop the ability to generate outputs that align with the patterns observed in the training data. The more data the model is trained on, the more precise it becomes in producing accurate outputs. The models developed by OpenAI (ChatGPT) and Google (Gemini) were released to the public to gather additional data through organic usage. In addition to these efforts of sourcing data from the public by natural user experiences, OpenAI and Google both gathered enormous amounts of data for their models to assimilate. OpenAI had roughly ten billion dollars of funding from Microsoft to train and develop their model. Google has access to even larger amounts of user data through their search engine, website ads, and many other data sources they own. This distinction in data-as-a-resource allowed OpenAI and Google to develop some of the leading models in terms of accuracy and performance. The potential creates possibilities for higher education mentoring and career guidance in STEM.

Human-centered approaches to mentoring in STEM fields play a pivotal role in guiding students from academic learning to professional careers. This form of mentorship is critical as it facilitates the practical application of theoretical knowledge, enabling mentees to acquire essential skills, attitudes, and professional networks essential for success in STEM (National Academies of Sciences, Engineering, and Medicine, 2020). According to National Academies of Sciences, Engineering, and Medicine (2020), effective mentorship not only fosters career and psychosocial development but also cultivates deep, impactful relationships that contribute to the holistic development of STEM professionals. Further, research by Atkins et al. (2020) highlighted that mentoring significantly aids the career planning process, especially for underrepresented minority students, by fostering a scientific identity and providing them with role models and opportunities for growth in research contexts. This support strengthens students’ self-identification as scientists and encourages diverse pathways into further STEM leadership, underscoring the need for tailored and research-focused mentoring approaches. These traditional mentoring methods are instrumental in developing the next generation of STEM leaders, making them an indispensable part of educational strategies aimed at enhancing career trajectories in these fields. However, as valuable as these types of mentoring relationships are, they are time and resource intensive.

While traditional human-centered mentoring in STEM fields has proven invaluable, generative AI offers a unique opportunity to bridge resource gaps, performing personalized tasks for students that can supplement human mentor availability (Neumann et al., 2021; Wollny et al., 2021). Scholars have highlighted the potential of AI to offer students more personalized career guidance, aligning with a changing labor market (Duan and Wu, 2024) as well as enhancing professional development in specific fields such as preservice teacher education (Lu et al., 2024). AI mentoring also has the potential to provide equitable access to resources, particularly through virtual mentoring and AI-driven platforms (Akiba and Fraboni, 2023). These platforms not only facilitate personalized career advising but also significantly elevate engagement and satisfaction among diverse and underserved student populations (Okado et al., 2023). As AI continues to change mentoring and career development, it has the potential to be a powerful tool for inclusivity and adaptability in learning environments.

However, despite these promising developments, there remains a critical gap in understanding how exactly mentors and mentees might integrate their current mentoring practices to make the most of generative AI. A critical area that remains underexplored is the integration of generative AI into the development planning aspects of IDPs. Students could utilize aspects of AI to tailor development plans to their own needs and career paths, while leaving time for more nuanced and human-directed activities. Addressing these gaps could enhance the functionality of platforms like myIDP and help mentors and mentees envision new ways to utilize the IDP process and make effective use of their mentoring time.

The purpose of this study is to investigate the potential of AI mentors to enhance myIDP practices for STEM graduate students. The research methodology comprises three key components: (1) development of a comprehensive set of AI-based prompts aligned with the current myIDP framework, (2) evaluation of these prompts by a diverse cohort of STEM graduate students, predominantly from underrepresented groups, and (3) collection of participant feedback on the efficacy of AI mentors and strategies for optimal integration with human mentorship. Participants tested the AI prompts while engaging with myIDP and provided insights on the strengths and limitations of AI mentorship. Additionally, they offered perspectives on effectively leveraging both AI and human mentors throughout the IDP process. The findings contribute to the emerging field of AI integration in STEM mentoring, highlighting potential benefits, identifying areas for improvement, and exploring avenues to empower underrepresented minorities in STEM.

## 2 Materials and methods

### 2.1 Materials

This study initially evaluated the LLM-supported technologies of Google Gemini and ChatGPT. To streamline the process, we decided to utilize only one platform. During the data collection phase of our study, Gemini rapidly enhanced its features, a development anticipated given Google’s extensive data collection, amassing over 7 billion data points daily for more than a decade (Sagiroglu and Sinanc, 2013). The vast resources available to Gemini were evident in its evolution during our prompt testing period. In our comparative tests, Gemini demonstrated more accurate real-time results and current responses compared to ChatGPT, pre-trained on data up to 2022. Furthermore, Gemini offered integration with Google products, such as Google Scholar, providing peer-reviewed literature to support its responses. Based on our architectural knowledge of LLMs and prompt testing outcomes, we concluded that Gemini was better suited for producing supportive results for myIDP guidance. The data for this study was generated using prompts created for myIDP, chosen due to its significant impact and extensive user base.

There are four major components in the myIDP framework: Assessment, Career Exploration, Create Plan, and Implement Plan. The research team, with diverse expertise in computer science, mentoring, myIDP, STEM career development, and research methods, collaboratively generated meaningful prompts by leveraging their varied knowledge. After initial testing of these prompts, the team refined the prompts to help graduate students use myIDP more effectively. The final prompts are shown in Table 1. The Assessment component included prompts to clarify unclear or unfamiliar assessment items for Skills (e.g., demonstrating workplace etiquette), Interests (e.g., negotiating agreements), and Values (e.g., independence vs. working alone). The Career Exploration component covered prompts for topics such as considering career fit, reading about careers, attending events and workshops, talking to people, and choosing a career path. The Create Plan component included prompts for creating goals related to career advancement, skill improvement, and project development. For the final component, Implement Plan, prompts were designed for building a mentoring team.

**Table 1:** Prompts with the sentiment analysis results.

myIDP	Examples of prompts	Number of uses	Number of comments	Average sentiment (−1 to + 1)
**Assessment**				0.37
Skills assessment (Optional)	What is [item name]? (e.g., What is [demonstrating workplace etiquette]?)	8	6	0.42
Interests assessment (Optional)	What is [item name]? (e.g., What is [negotiating agreements]?)	8	6	0.43
Values assessment (Optional)	What is [item name]? (e.g., What is [independence]? What is [working alone]?)	10	5	0.23
**Career exploration**				0.26
Consider career fit (Choose at least one prompt)	What are some ways to know if I would be a good [job title]?	17	12	0.32
	[Talk about your top values first]. Could I pursue a career in [career path]?	5	3	0.78
Read about careers (Choose at least four prompts)	What is a typical workday as a [job title]?	17	12	0.03
	What is the average salary for a [job title] in [career path] this year?	16	11	0.13
	What job searching websites can I use to find job postings in [career path]?	10	11	0.24
	What skills and qualifications are typically required for careers in [career path]?	10	4	0.19
	What is the demand like for jobs in [career path]?	8	4	0.23
	What are some potential career growth opportunities or advancement prospects in [career path]?	4	2	0.5
	What are the main challenges or drawbacks associated with careers in [career path]?	7	4	0.36
	Are there any specific certifications or additional training that can enhance job prospects in [career path]?	5	3	0.42
	Can you suggest any resources or websites to explore for further information on careers in [career path]?	4	2	0.6
	What does the future hold for jobs in [career path]?	1	1	0.63
	Are there any notable trends or emerging areas within [career path] that might impact future job prospects?	1	0	NA
Attend events and workshops (Choose at least three prompts)	What are some annual or regional events in [city/locale] to assist in [given goal]?	6	2	0.21
	What are some upcoming events or workshops related to [career path] in [city/locale]?	13	9	0.3
	Can you recommend any conferences or industry-specific events that are beneficial for someone interested in [career path]?	11	6	0.15
	Are there any scholarships, grants, or funding opportunities available for attending career-related events or workshops in [career path]?	10	6	0.36
	How can attending events or workshops help me gain insights and network within [career path]?	6	2	0.33
	How can I make the most out of attending events or workshops to enhance my career prospects in [career path]?	3	3	0.33
	Can you suggest any resources or websites where I can find information about career-focused events and workshops in [career path]?	6	3	0.38
Talk to people (Choose at least four prompts)	Where can I find people to ask questions about [career path] topics?	6	3	0.15
	What are some effective strategies for networking in [career path]?	12	5	0.46
	How can I identify and approach professionals in [career path] for informational interviews?	11	5	0.21
	What are some key questions to ask during an informational interview?	12	5	0.4
	How can I make a positive impression and build connections through networking events or online platforms?	8	4	0.22
	Can you provide tips on following up and maintaining relationships after networking events or informational interviews?	9	5	0.32
	How important is it to establish a personal brand or online presence for networking purposes?	6	4	0.09
	Are there any common networking mistakes to avoid?	7	3	0.21
	How can I leverage social media platforms for professional networking in [career paths]?	1	0	NA
	Are there any specific resources or websites to help me find networking opportunities in [career path]?	3	1	0.44
	Can you suggest some professionals in [career path] in [city/institution], with whom I can connect to gain insights into a career in [career path]?	5	2	0.36
Choose a career path (Required)	[Talk about your long-term goal first]. What transition experience do I need to reach this long-term goal? [You may have follow-up questions about the suggestions from AI]	18	8	0.26
**Create plan**				0.37
Career advancement goals (Choose at least one prompt)	As a [role], what are some SMART career advancement goals [in the next X years]?	11	7	0.23
	[Describe your current status]. This year, I want to [list the career advancement areas you want to improve this year]. Do you think my plan is feasible?	7	4	0.47
	[Describe your current status]. What are my SMART goals to improve [your target career advancement area this year]? [You may have follow-up questions about the suggestions from AI]	2	1	0.42
Skill goals (Choose at least two prompts)	How [SMART metric] is this goal for [identity] on a scale of 1 to 10 with 10 being unlikely? [Goal]	2	0	NA
	What are some SMART skill goals for [identity] in [concentration/major]?	6	4	0.43
	What are some SMART skill goals for [career path]?	8	4	0.48
	What are some SMART skill goals for becoming a [career path]?	9	2	0.57
	[Describe your current status]. This year, I want to [list the skills you want to improve this year]. Do you think my plan is feasible?	5	1	0.46
	[Describe your current status]. What are my SMART goals to improve [your target skill this year]? [You may have follow-up questions about the suggestions from AI]	8	3	0.35
Project goals (Choose at least two prompts)	How [SMART metric] is this goal for [context] on a scale of 1 to 10 with 10 being unlikely? [Goal]	2	2	0.31
	What are some SMART project goals for [context] in [concentration/major]?	7	3	0.27
	What are some SMART project goals for [career path]?	10	6	0.32
	What are some SMART project goals for becoming a [career path]?	10	5	0.32
	[Describe your current status]. This year, I want to [list the project areas you want to improve this year]. Do you think my plan is feasible?	3	1	0.53
	[Describe your current status]. What are my SMART goals to improve [your project area this year]? [You may have follow-up questions about the suggestions from AI]	4	1	0.42
**Implement plan**				0.52
Mentoring team (Choose at least three prompts)	What are the key qualities or characteristics to look for in a mentor?	14	5	0.59
	How can I identify potential mentors who align with my career goals and interests?	7	3	0.39
	What are some effective strategies for approaching and initiating conversations with potential mentors?	7	2	0.64
	How can I evaluate whether a mentor is a good fit for my development needs?	10	3	0.56
	What are some common challenges in mentoring relationships and how can I address them proactively?	8	2	0.44
	What are the benefits of having a diverse mentoring team?	2	1	0
	How can I maintain and nurture my relationships with mentors over the long term?	4	2	0.4
	What are some effective ways to communicate my expectations and goals to my mentors?	7	0	NA
	How can I make the most of each mentoring session or interaction?	4	2	0.57
	[Describe your career goals or areas you want to improve]. Are there any specific industries or professional networks where I can find mentors in my field?	1	0	NA

Our research participants had the freedom to interact with AI mentors using the suggested prompts. To prevent overwhelming the participants or diminishing their motivation and interest in working with AI mentors, we asked them to test some of the prompts that they found useful when working on myIDP. Providing comments for each prompt was not required, which may result in the number of comments being smaller than the number of uses. Additionally, instructions in the first column, such as “Choose at least three prompts,” were designed for the research participants. We encouraged readers to use any AI prompts in the table they found useful.

To integrate Google Gemini as an AI-mentor within the myIDP framework, participants were asked to use the prompts we generated (see Table 1) while navigating through the myIDP process. The prompts we generated were designed to help mentees acquire critical, up-to-date information, regarding their career development, which the myIDP portal cannot efficiently provide. For instance, the prompt “What is a typical workday as a [job title]?” could be used when mentees were navigating through the process of “Read about Careers” in myIDP. By using the generated prompts to ask Google Gemini questions aligned with the different aspects in myIDP, the AI mentor can be effectively integrated with the myIDP framework. The process of providing feedback on their experience with the AI-mentor and prompts will be addressed in the next section.

### 2.2 Methods

#### 2.2.1 Data collection and sample

After the study was approved by the Institutional Review Board at the authors’ university (Protocol #HM20028450), we recruited participants via the university’s daily events newsletter as well as directed communication via the School of Engineering and Career Services. Starting with the myIDP website, participants were asked to integrate the provided prompts into their myIDP process, then provide feedback through a survey about their experiences using Gemini as their AI mentor. To help the participants with the process, we provided a tutorial that walked them through the myIDP process as well as how to use Google Gemini as an AI mentor with the prompts. Participants provided their comments on the prompts they tested and the responses from the AI mentor along with the Google Gemini public shared links. After completing the prompts testing, participants were asked to complete open-ended questions of their thoughts on the strengths and concerns of using the AI mentor. Participants each received a $100 gift card to compensate for their participation.

This study involved 18 participants, all of whom were from STEM fields, as defined by the U.S. Department of Homeland Security’s STEM Designated Degree Program List (U.S. Department of Homeland Security, 2023), which includes health-related disciplines. Of these, 15 were full-time graduate students in the United States, and three were visiting students from other countries. Half of the participants were in the initial stages of their master’s programs, while the others were at various stages of their doctoral studies. These students were pursuing careers across a broad spectrum of fields, aiming to make significant contributions in both the public and private sectors. The demographic composition of the participants reflected a focus on including underrepresented groups in STEM. Specifically, 15 of the participants identified as women or non-binary/genderqueer, and 4 identified as Black or Hispanic/Latino. A total of 16 participants were classified as underrepresented minorities, with the remaining two being Asian men. Additionally, eight participants were first-generation college students, and six were international students. In terms of familiarity with the IDPs, while only seven participants had prior experience, all received training on the myIDP tool before participating in the study. Background information of the participants can be found in Table 2.

**Table 2:** Participants’ background information.

ID	Degree	Year	Career goal	Gender	Race/ethnicity	Age	First-gen	International student	IDP experience
S1	Master’s	First year	Biotechnology industry scientist	Woman	Multiracial	18–23	No	No	No
S2	Master’s	First year	Government conservation organization	Nonbinary /gender queer	Hispanic or Latino	18–23	Yes	No	No
S3	Doctoral	sixth year	Postdoctoral fellowship	Woman	White	30–39	No	No	Yes
S4	Doctoral	Third year	Occupational therapist	Man	Black	24–29	Yes	No	No
S5	Doctoral	Third year	Academia	Woman	White	24–29	No	No	Yes
S6	Doctoral	Fourth year	Material scientist	Man	Asian	24–29	Yes	Yes	Yes
S7	Master’s	First year	Fertility or medicine	Woman	Asian	18–23	No	No	Yes
S8	Master’s	First year	Software engineering	Man	Asian	24–29	Yes	Yes	No
S9	Master’s	First year	Public health related	Woman	Asian	30–39	Yes	Yes	No
S10	Doctoral	Fourth year	Doctor	Woman	Black	24–29	No	No	No
S11	Doctoral	First year	Community College Instructor	Woman	White	30–39	No	No	Yes
S12	Doctoral	Second year	Private industry or NGO	Woman	Black	30–39	No	No	Yes
S13	Doctoral	Third year	Private industry	Woman	White	30–39	Yes	No	Yes
S14	Doctoral	Third year	Clinic work and academia	Woman	White	24–29	Yes	No	No
S15	Master’s	First year	Ecology research	Nonbinary /gender queer	White	18–23	No	No	No
S16	Master’s	Second year	Counselor	Woman	Asian	24–29	No	Yes	No
S17	Master’s	Second year	Counseling psychologist	Woman	Asian	24–29	Yes	Yes	No
S18	Master’s	Third year	Helping professionals work	Woman	Asian	24–29	No	Yes	No

#### 2.2.2 Data analysis

Participants’ comments to selected prompts around Assessment, Career Exploration, Create Plan, and Implement Plan were assessed with sentiment analysis using VADER from the nltk Python package. This sentiment analysis method is a supervised program of mapping text-by-word tokenization to the speakers’ feelings and emotions (Devika et al., 2016). Comments were tokenized into individual words and assigned a score from −1 being most negative, 0 being neutral, and + 1 being most positive as they have been previously defined in the VADER lexicon. The lexicon is pre-trained and provided openly by the ntlk Python package.

Due to the limited number of responses in our sample, we examined the aggregate sentiment to have a loose idea of how participants favored our prompts. Through running our sentiment analysis on participant comments, we noticed the algorithm has approximately 90% accuracy in rating the sentiment of the comments. Only the categorical impressions, the average of all prompt aggregate sentiments within the category are mentioned below, but we provide the more detailed sentiment analysis results in Table 1. Results were analyzed in conjunction with a word cloud to better understand the most pertinent comments. In addition to the sentiment analysis, participants’ comments were analyzed using the thematic analysis approach to identify recurring and important perspectives.

Participant responses regarding the strengths and concerns of AI mentors were also analyzed using the qualitative data analysis software packages, Atlas.ti and MAXQDA. A thematic analysis approach was employed to systematically identify and categorize recurring themes within the data. The process began with familiarization, where researchers reviewed all responses to gain a comprehensive understanding of the content. Key points and significant statements were then identified and coded, marking relevant segments of the data. These codes were grouped into potential themes, reflecting broader patterns and advantages and concerns identified by the participants. Themes were subsequently reviewed and refined to ensure they accurately represented the data. Finally, clear names were assigned to each theme to encapsulate their meaning as understood by the researcher. This methodological approach provided an in-depth analysis of participant responses, highlighting both the strengths and concerns associated with their engagement with AI mentors.

To ensure the reliability of our thematic coding, we employed a multi-coder approach, enhancing the reliability of our findings through intercoder agreement (Creswell, 2012). Each coder independently developed a set of codes through team discussions and reflexive reading of the participant responses. These codes were then synthesized into overarching themes, which highlighted higher-level advantages and disadvantages. The team collectively reviewed and discussed these themes to gain a comprehensive understanding of the potential use of AI for mentoring purposes. This collaborative process aimed to minimize individual bias and enhance the reliability of our qualitative analysis.

## 3 Results

### 3.1 AI prompts

#### 3.1.1 Assessment

Comments for the assessment prompts were relatively few. The assessment categorical sentiment was moderately positive (0.37). Most participants had somewhat positive experiences using the prompts to clarify certain concepts. The participants found it helpful in explaining terms and also in providing useful insights on what they asked the AI mentor. For example, one participant asked the AI mentor the meaning of “keeping up with current events” and thought that the information was not only helpful but also provided useful insights as a growing researcher.

#### 3.1.2 Career exploration

Overall, participants reported positive experiences with the prompts for career exploration. Career categorical sentiment was the least positive (0.26). Among the comments, many of them affirmed that the AI mentor could provide helpful feedback on their career fit by using the prompt, “What are some ways to know if I would be a good [job title]?” More than one participant thought that the responses from the AI mentor were thorough.

Participants also shared slightly positive experiences (0.22) with asking AI mentors questions regarding the prompts of “Read about careers.” For example, by using the prompt “What is a typical workday as a [job title]?” The AI mentor could provide useful information about participants’ interested career paths. Participants also commented that the AI mentor’s responses were similar to their past experiences. One participant mentioned that the AI mentor provided different examples of workplaces and workday circumstances, which was helpful. However, some participants maintained skeptical attitudes against the responses from the AI mentor based on their understanding of the career path.

When asking the AI mentor questions about “attending events and workshops,” participants found that the AI mentor often lacked specific information for their local areas, though still provided some useful items for consideration (0.29). One of the participants commented that “I first asked [annual or regional events] about a specific city and Gemini was not able to generate the events.” Sometimes, if the question did not include the current year (i.e., 2024) for the conference or events, Gemini provided information for the previous year (i.e., 2023). Also, across different prompts, some participants found that the AI mentor was not providing related links regarding job-searching websites, resources, or scholarship opportunities. One participant noted, “having links to the sites would be better so I do not have to do another search.” Another negative comment was that the AI mentor tended to provide generic responses, ignoring the specific information participants indicated in their prompts. For example, when asking, “What are some potential career growth opportunities or advancement prospects in [career path]?” One participant claimed that “I was hoping it would list some internships, but it instead was very broad.”

#### 3.1.3 Create plan

For the prompts within “Create Plan,” most of the participants provided moderately positive feedback on the prompts for SMART goals (0.37), including SMART career advancement goals (0.33), skill goals (0.45), and project goals (0.33). Participants mentioned that the AI mentor was able to provide examples of SMART goals, which they would like to add to their plans. For example, when asking questions such as “what are some SMART project goals for becoming a senior software engineer,” the participant commented that “The answer helps me understand, in order to achieve my career goal, what to do at the moment.” However, one participant found that the AI mentor did not recognize what SMART goals are. Also, another participant thought that the SMART goals suggestions from the AI mentor were poor due to “few insights of the industry.”

#### 3.1.4 Implement plan

Participants provided the most positive feedback for prompts related to seeking mentors (0.52). They found value identifying key qualities of mentors, questions to be asked when seeking mentors, and how to interact with potential mentors. For example, several participants appreciated the AI mentor’s response to the question about “key qualities to look for in a mentor position,” which helped them understand how to identify a good mentor. Participants also learned some critical aspects to consider when seeking human mentors, such as mentoring philosophy. However, one participant mentioned that the response for the mentor evaluation prompt was too generic and lacked depth.

#### 3.1.5 Additional feedback from international students

##### 3.1.5.1 US-centric responses

When asking questions, it’s crucial to specify the country to avoid receiving US-centric answers. For example, even when using a Virtual Private Network (VPN) service, the responses often defaulted to U.S. users. Indicating the country name, even in the local language, may result in more accurate and contextually relevant responses.

##### 3.1.5.2 Career information accuracy

A participant sought career guidance for Country A, but the AI mentor provided information and resources from Country B due to the two countries sharing a similar language. Additionally, some career information, such as licensing exams, is often incorrect for countries outside the U.S.

##### 3.1.5.3 Cultural sensitivity in communication

The suggested email templates for reaching out to potential mentors can sometimes be too aggressive and fail to consider cultural differences. This can be particularly problematic in some countries where a more formal or respectful approach is preferred.

### 3.2 Strengths of AI mentoring

#### 3.2.1 Immediate response

Most of the participants appreciated the immediate responses from the AI mentor. Compared with human mentors, the AI mentor allowed them to navigate through the myIDP process without waiting for unclarified questions. Several participants shared their past experiences of waiting for responses from their human mentors during the process, which were in strong contrast to experiences with the AI mentor.

#### 3.2.2 Up-to-date information

Some participants affirmed that the AI mentor could provide up-to-date information. Nevertheless, some participants also mentioned some limitations. For example, the AI mentor was not able to provide information regarding upcoming or local events. One participant also acknowledged that the AI mentor is not always trained on up-to-date datasets, which caused its lack of knowledge on the most recent event. On the other hand, two of the participants mentioned that the information provided by the AI mentor was the same as that of their human mentors. Although the participants recognized this as not up-to-date information, it showed that the AI mentor shared the same knowledge as human mentors.

#### 3.2.3 Access to multiple AI mentors

In Google Gemini, users can choose different versions of responses. Four participants thought this function was helpful for them to gain various points of view on the same questions. However, seven participants thought that the AI mentor was just rephrasing the same concepts, and the responses looked generally the same. One participant also expressed concerns about creating biases by frequently selecting preferred versions of responses (“I think it is neat that it provides multiple answers, but I feel nervous it would play into my own biases if I kept refreshing for one that I liked better.”).

#### 3.2.4 Enhanced ownership of career development

Nine participants expressed positive experiences with taking ownership when they interacted with the AI mentor. One participant expressed that the process of interacting with the AI mentor allowed mentees to take the lead in the direction of the conversation. Another participant also mentioned that unlike human mentors, the AI mentor allowed users to ask many questions whereas that person may not feel quite as comfortable asking their human mentor 20 random questions in a row. One participant stated that by interacting with the AI mentor, it increased mentee’s confidence because the AI mentor did not tell the participant what to do but only provided recommendations. In contrast, three participants expressed that they did not feel “ownership” during the process. One participant argued that the information provided by the AI mentor can also be acquired through Google search.

#### 3.2.5 Time savings

When it comes to the advantage of affecting time for human mentors and mentees, most of the participants agreed that the AI mentor helped them save time from gathering information on their own. One participant mentioned that using the AI mentor can help narrow down questions for human mentors that AI could not answer specifically.

#### 3.2.6 Other

The feedback from the participants addressed additional advantages of using the AI mentor. First of all, the flexibility of the AI mentor. Six participants praised how flexible the AI mentor is during the process. Users can ask anything without hesitation. In addition, for students who do not have human mentors or who feel uncomfortable talking to human mentors, AI mentors can be helpful. Second, the AI mentor can help bridge the mentorship between mentees and human mentors. Some participants suggested that the responses from the AI mentor can serve as a starting point for thinking of questions to ask their human mentors or to help spark an idea. Third, the process of using the AI mentor enhanced their myIDP experience. Participants mentioned that the process promoted them to review their myIDP components and help them set career goals.

### 3.3 Concerns of AI mentoring

#### 3.3.1 Misinformation

Participants generally felt that the AI mentor provided accurate information, with the majority noting its overall reliability. Specifically, participants mentioned that the AI was overall accurate, and others noted that it was mostly accurate with occasional inaccuracies or minor contradictions. For example: “When I asked about the demand for neuroscience researchers, Gemini first said that the demand was high, but then a couple of paragraphs later, said that there are more PhD graduates than there are faculty positions (which is true).” However, some participants felt that the AI’s advice was too general to be of significant use. One participant noted the AI’s consistency with guidance from human mentors, and another found the AI to be a good starting point for further exploration and research.

#### 3.3.2 Bias

Gemini’s learning process relies on real-world data, which may contain inherent bias. As a result, its responses sometimes reflect stereotypes and discriminatory information. For instance, when developing prompts for the research project, our team observed a disparity in the suggestions for STEM career paths provided to doctoral students based on gender. When offering career advice to a mom, the AI mentor emphasized factors like “flexibility,” “balancing family responsibilities,” and “supporting working moms.” On the other hand, when advising a dad, the AI mentor focused on more general aspects such as “interests and passions,” “family situation,” and “financial goals.”

Participants did not report any instances of discriminatory or biased responses from the AI mentor. However, there were some general concerns about the potential for bias listed here and in other sections. One participant noted that while a human mentor might exhibit more bias based on physical appearance, “technology has advanced tremendously and I did just give a lot of my personal information through these questions so it could be building a profile of what they think I am.” This raises concerns about data usage for algorithmic purposes, creating the potential for bias. Another participant expressed a wish to have asked more questions about their career goals in relation to their chronic health disability, to see if the AI could offer relevant advice or if it might show ableist tendencies. These reflections show participant worries about bias and data privacy, even in the absence of explicit biased responses from the AI.

#### 3.3.3 Privacy

Participants expressed a range of feelings about using AI mentors, particularly concerning privacy. Some participants felt there was a balance between privacy concerns and the personalization available to them while using AI mentors, while others were cautious about sharing personal information with AI mentors. One participant compared their lack of privacy concerns to their usage of social media, indicating a similar level of comfort with information they shared on social networking and social media platforms with what the AI platform knew about them. Conversely, some participants were worried about the transparency of data storage and usage, with some expressing general concerns about data privacy. One participant summed up the contradiction between needing to supply personal information to an AI mentor for useful feedback and privacy concerns in this way: “I do worry about privacy and selling data. I think turning off tracking (or limiting it to the session) could help. On the one hand, Gemini cannot get to know me like a human mentor could and provide advice accordingly. On the other hand, I do not want to give Gemini enough personal information for it to give specific advice.”

#### 3.3.4 Equity

Participants’ responses revealed several challenges and perspectives regarding access to AI mentors within marginalized communities. The predominant concern was internet and technology access, highlighting the digital divide as a significant barrier to AI mentor usage. Some participants highlighted the continued importance of human mentors, suggesting that despite the advantages of AI, the value of human connection and personalized guidance remains critical. Another important issue raised was internet literacy, as not everyone currently possesses the skills to evaluate online information effectively. “Any lack of internet literacy may lead someone to believe anything they read online. I also think that those who are better able to communicate with AI are more likely to get more accurate answers.” Language barriers were also noted by one participant as a potential obstacle. One participant emphasized the necessity for better access for people with disabilities, indicating a need for inclusive design. Interestingly, one participant felt that AI mentors could possibly be more accessible to marginalized individuals than human mentors, offering a unique viewpoint on the potential benefits of AI mentorship. Overall, while AI mentors might offer potential benefits, participants noted significant challenges such as technological barriers including access and literacy, as well as inclusivity which need to be addressed to ensure equitable access for all communities.

#### 3.3.5 Algorithmic influence

Participants expressed various concerns about the unknown working nature of AI, but many chose not to answer the question or did not understand the question, suggesting a lack of familiarity with AI. Three participants viewed AI as a complementary tool to human mentors, emphasizing its supplementary role, and another three called for more transparency in AI technology development. Concerns about the vulnerability of less experienced users were noted by two participants, while three participants worried about the potential for AI to provide false information.

#### 3.3.6 Mentoring support

Participants overwhelmingly agreed that AI cannot replace real human mentoring, though many recognized that AI could serve as a valuable supplement to human mentoring. The efficiency of AI, offering quick and accessible guidance, was noted as a significant benefit. Some participants also saw AI as a great starting point for career development conversations, particularly for youth. When access to human mentors is limited, one participant also noted that AI mentoring can also be particularly useful.

#### 3.3.7 Other

When participants were given an open-ended opportunity to address personal concerns about the use of AI mentors, one unique concern was becoming over reliant on technology, potentially leading to a loss of soft skills like communication, mentoring, and connection. Some participants emphasized the need for training in AI usage, such as tutorials or guidance on how to use AI effectively as a mentor in order to address literacy concerns and help mentees treat AI as a tool and not as a replacement for human mentors.

### 3.4 Human-AI mentoring

Two hybrid human-AI mentoring models emerged from participants’ input: the *Sequential Integration Mentorship Model* and the *Concurrent Collaboration Mentorship Model*. These models leverage the unique strengths of both human and AI mentors, creating a synergistic approach to mentoring that enhances the developmental process for mentees.

#### 3.4.1 Sequential integration mentorship model

This hybrid model organizes the mentorship process into distinct, sequential phases, leveraging the unique strengths of human and AI mentors at different stages. Initially, *AI as Initial Point of Contact* engages with mentees by offering broad, general perspectives and foundational knowledge on the mentee’s career path. This is particularly beneficial when direct human mentorship is not immediately available, ensuring no delay in the mentee’s developmental process. Following this groundwork, *Transition to Human Mentorship* occurs, with human mentors stepping in to deliver detailed, personalized guidance tailored to the specific needs identified during the initial phase. This seamless handover ensures continuity and depth in mentoring. After each mentorship phase, *IDP Refinement Feedback* is provided by human mentors based on their interactions with mentees. This feedback is leveraged to *enable AI to offer more customized information*, specifically aimed at mapping out a more tailored development plan. This iterative improvement process ensures that the AI’s contributions are finely attuned to the evolving needs and goals of mentees, enhancing the precision and effectiveness of the mentorship.

#### 3.4.2 Concurrent collaboration mentorship model

This hybrid model features continuous and simultaneous collaboration between human and AI mentors throughout the entire mentorship process. This model fosters a dynamic integration of efforts, with AI and humans contributing in real-time without clear boundaries between their roles. AI provides *Immediate Assistance* (rapid, general advice, quick feedback, and emergency support), managing real-time information flow and addressing straightforward queries efficiently. Additionally, AI compiles resources, crafts structured plans, sets SMART goals, and stimulates creative thinking for career and mentorship development. On the other hand, human mentors offer *Personalized and Emotional Support*, utilizing their real-world experience to address complex personal or professional challenges, ensuring the mentorship is empathetic and practical. Human mentors also *Contextualize AI Data* by interpreting and adjusting AI-generated data and recommendations to fit the unique contexts of each mentee. This *Synergistic Interaction* between human and AI mentors enriches the mentorship process by providing a holistic view, diversifying resources, and cross-referencing each other’s inputs to ensure comprehensive development. By leveraging both AI and human insights, the mentorship experience continuously evolves and adapts to meet the mentees’ needs effectively.

## 4 Discussion

**Figure 1:**
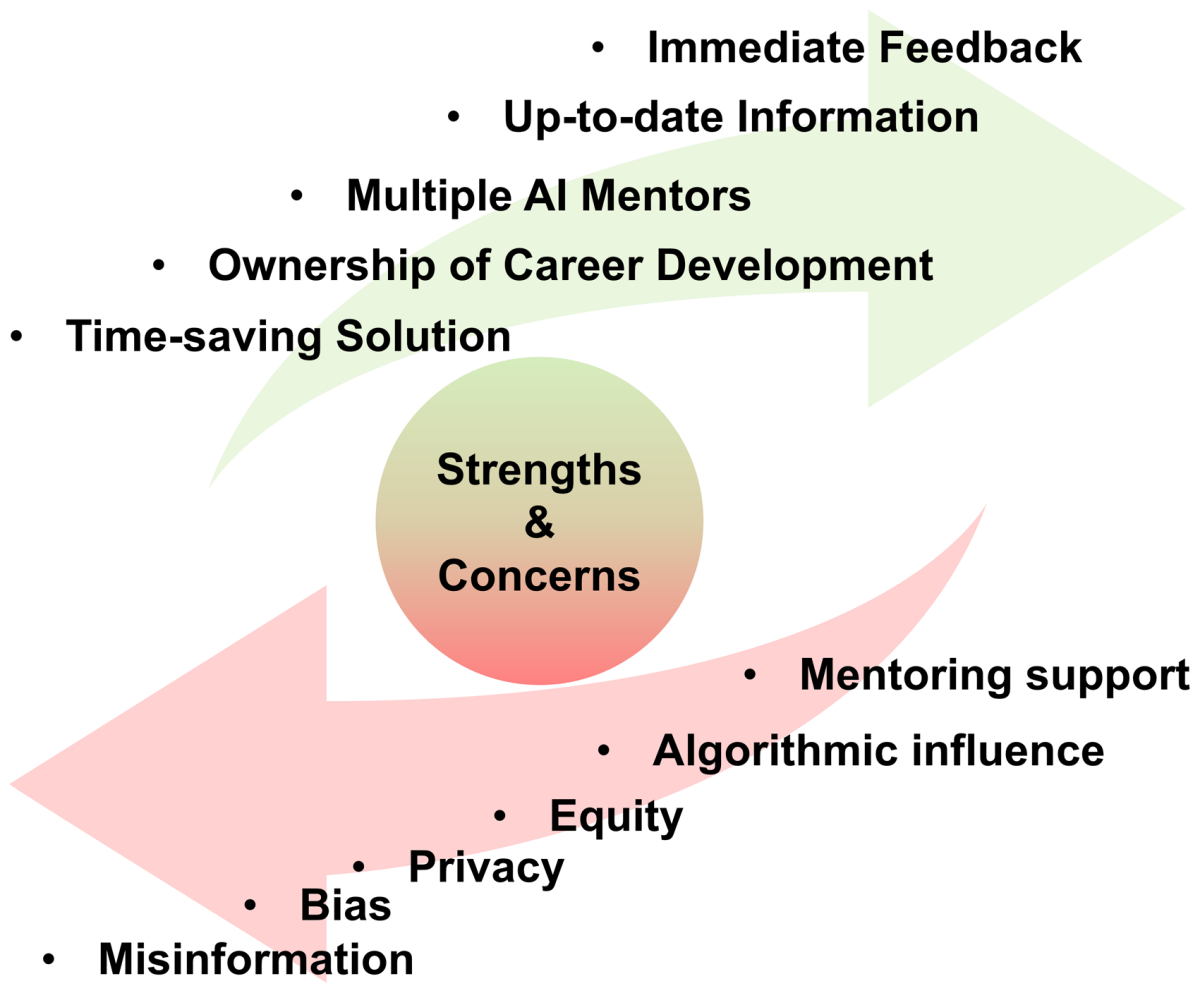
Strengths and concerns of using AI mentors.

In light of recent mandates from the U.S. National Science Foundation (NSF) under the CHIPS and Science Act of 2022, which require IDPs for all NSF-funded graduate students (National Science Foundation, 2024), this study aims to optimize career development processes using IDPs. It explores integrating AI support within the myIDP framework to empower STEM graduate students, particularly those from underrepresented minority groups, by providing personalized career guidance. To achieve this, the research team developed a series of AI prompts tailored for use within myIDP. Eighteen STEM graduate students, mostly from underrepresented minority backgrounds, were trained to interact with AI mentors. Their interactions, along with comments and feedback, were analyzed using sentiment and thematic analysis, shedding light on the strengths and concerns associated with AI mentorship (Figure 1). The findings also suggest two hybrid models for human-AI collaborative mentoring, where both agents work synergistically to provide personalized guidance throughout the IDP journey.

### 4.1 Effectiveness of AI mentoring

**Figure 2:**
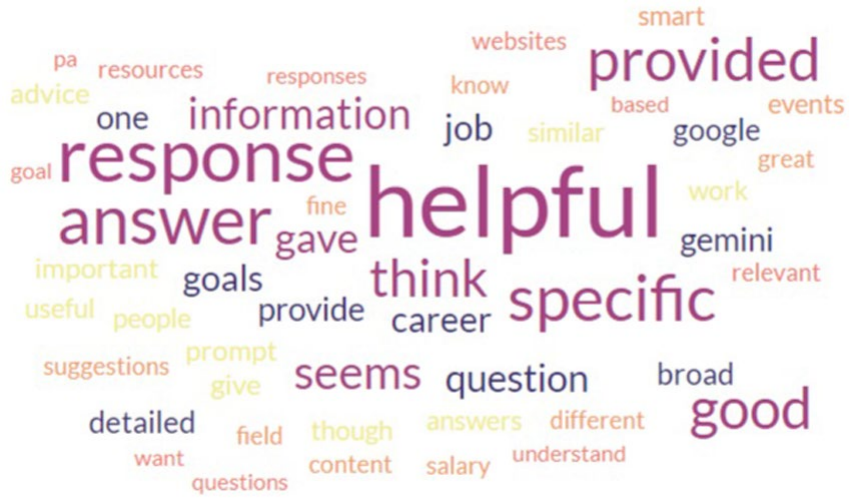
Word cloud of feedback from participants after using AI prompts to support career development.

Based on the comments for each prompt from the participants, we found that most participants had positive experiences when using the prompts for their myIDP process (Figure 2). However, AI responses may have been perceived as less helpful in some categories than others because of nuanced areas requiring more human interactions beyond current capacities of AI models. Further, some participants commented that the responses from the AI mentor were rather generic. This is often due to the limited information provided in a short prompt. When using the AI mentor, it is important to ask follow-up questions to obtain more contextually accurate or sophisticated responses. Some participants’ experiences did improve by asking further questions to the AI mentor, specifically mentioning that the AI mentor recommended contextual information in later prompts. Nevertheless, it is also important to acknowledge that these are highly likely one of the limitations of the AI mentor at the stage of AI development during this study.

We also found that certain prompts were selected more often by our participants (Table 1). The tendency likely stems from the attributes of requesting information. Popular prompts such as “What is a typical workday as a [job title]?” and “What is the average salary for a [job title] in [career path] this year?” are the ones that require up-to-date information. We hypothesized that these would be the types of questions the AI mentor performs well with, however, sentiment analysis gave us more insight into which prompts were generally more useful. Prompts such as “[Talk about your top values first]. Could I pursue a career in [career path]?” and “What are the key qualities or characteristics to look for in a mentor?” yielded highly positive sentiments among participants. On the other hand, some prompts were rarely selected such as “Are there any notable trends or emerging areas within [career path] that might impact future job prospects?” or “How can I leverage social media platforms for professional networking in [career paths]?”

When thinking of the strengths of using the AI mentor, flexibility is the most significant one. Most participants appreciated the immediate response from the AI mentor, the flexible time, and the free space it provided to ask questions. Mentees do not need to wait for responses when they encounter questions during the myIDP process. They can ask numerous questions to the AI mentor without hesitation. The flexibility of the AI mentor not only provides a convenient approach for mentees to solve their questions or obtain information, but also enhances their experiences when developing career plans. Another major advantage of using the AI mentor is that it can serve as a brainstorming tool during the myIDP process. Their comments and feedback suggest that using the AI mentor helped them rethink their original plan or provided a feasible starting point through details and perspectives that were previously missed. By exploring information and setting SMART goals with the AI mentor, it enhanced their myIDP process by clarifying terms in myIDP or providing sparkling ideas about their career development plan. In turn, this information can allow a human mentor to spend more time focusing on the nuanced and abstract aspects of career development during meetings.

Integrating AI mentors into graduate education presented several concerns for participants, including the accuracy and reliability of AI-generated advice, which often requires human feedback for accuracy and contextual appropriateness. Privacy and data security issues also demand policies and transparent data usage to build trust. While AI can offer quick, data-driven insights, there was a fear among participants that using AI in this way could lead to an overreliance on technology and a failure to maintain vital soft skills like communication. There is also a need for ongoing monitoring of the quickly growing field of AI, coupled with user training on AI’s capabilities and limitations. All these concerns led many participants to conclude that AI mentoring was best seen as a tool not as a replacement for human mentors. Their comments pointed toward an approach that fosters collaboration between human and AI mentors, leveraging the strengths of both to enhance the mentoring process.

### 4.2 Ethical and equity implications of AI mentoring

The integration of AI into graduate education mentorship also raises significant ethical and equity concerns that must be considered and addressed to ensure fair and inclusive outcomes. One of the primary ethical challenges is the concern for algorithmic bias, where AI systems may inadvertently perpetuate existing societal biases, leading to unequal treatment of mentees based on race, gender, socioeconomic status, or other marginalized identities. This bias could manifest in AI-generated advice that disproportionately favors certain groups while disadvantaging others, exacerbating existing inequalities within academic and professional environments. Additionally, there is a risk that AI mentors may lack cultural sensitivity and fail to account for the unique experiences and needs of diverse mentees, further widening the gap in mentoring quality. Some participants noted that the responses failed to incorporate geographically diverse answers to the prompts, particularly international recommendations. To mitigate these risks, it is essential to implement bias detection and correction mechanisms within AI systems, ensuring that they operate fairly and equitably. More importantly, to remind users to carefully assess the answers and not take each answer for granted, such as bringing the answers to discuss with a more experienced human mentor. Ethical oversight and transparency in AI development and deployment are crucial, allowing for continuous evaluation and improvements of these systems. Ensuring that AI mentors are accessible to all students, regardless of their background, and that they complement rather than replace human mentors, can help create a more equitable and inclusive mentoring environment.

### 4.3 Hybrid human-AI mentoring models

**Figure 3:**
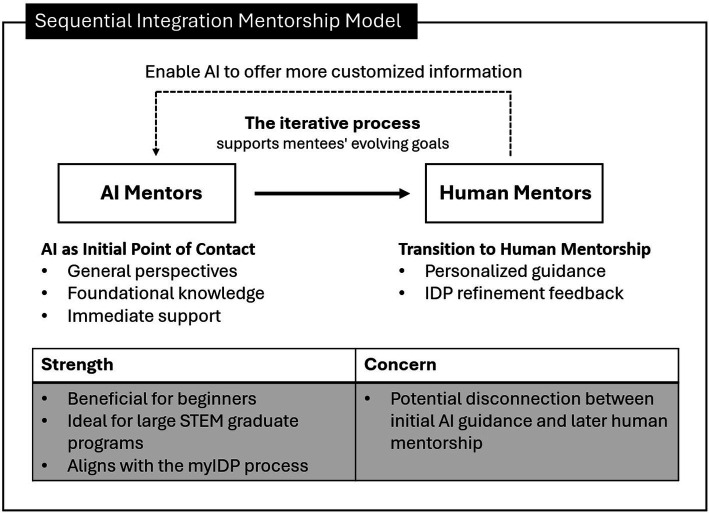
Sequential Integration Mentorship Model showing human-AI mentorship flow.

Our study addresses a critical gap in understanding the integration of human mentors and generative AI into the STEM career planning process. The findings reveal two conceptual human-AI mentoring models: the Sequential Integration Mentorship Model and the Concurrent Collaboration Mentorship Model. The Sequential Integration Mentorship Model (Figure 3) organizes the mentorship process into distinct, sequential phases. Initially, AI serves as the initial point of contact, engaging with mentees by offering broad, general perspectives and foundational knowledge on their career path. This phase is particularly beneficial for beginners, such as first-year graduate students, who may lack fundamental knowledge and feel pressured to ask questions. AI provides an opportunity for self-exploration without the immediate need for human interaction. Following this groundwork, there is a transition to human mentorship, where human mentors step in to deliver detailed, personalized guidance tailored to the specific needs identified during the initial phase. This transition ensures continuity and depth in mentoring. After each mentorship phase, IDP refinement feedback is provided by human mentors, enabling AI to offer more customized information specifically aimed at mapping out a more tailored career development plan. This model aligns perfectly with the myIDP framework, where self-assessment and self-reflection are supported by AI, followed by human mentors providing further feedback and customized updates. It is ideal for large programs with high mentee volumes or for mentees needing foundational knowledge before personalized guidance. However, a potential weakness is the disconnect that may occur between the initial AI guidance and later human mentorship, which may not be ideal for mentees who require ongoing support.

**Figure 4:**
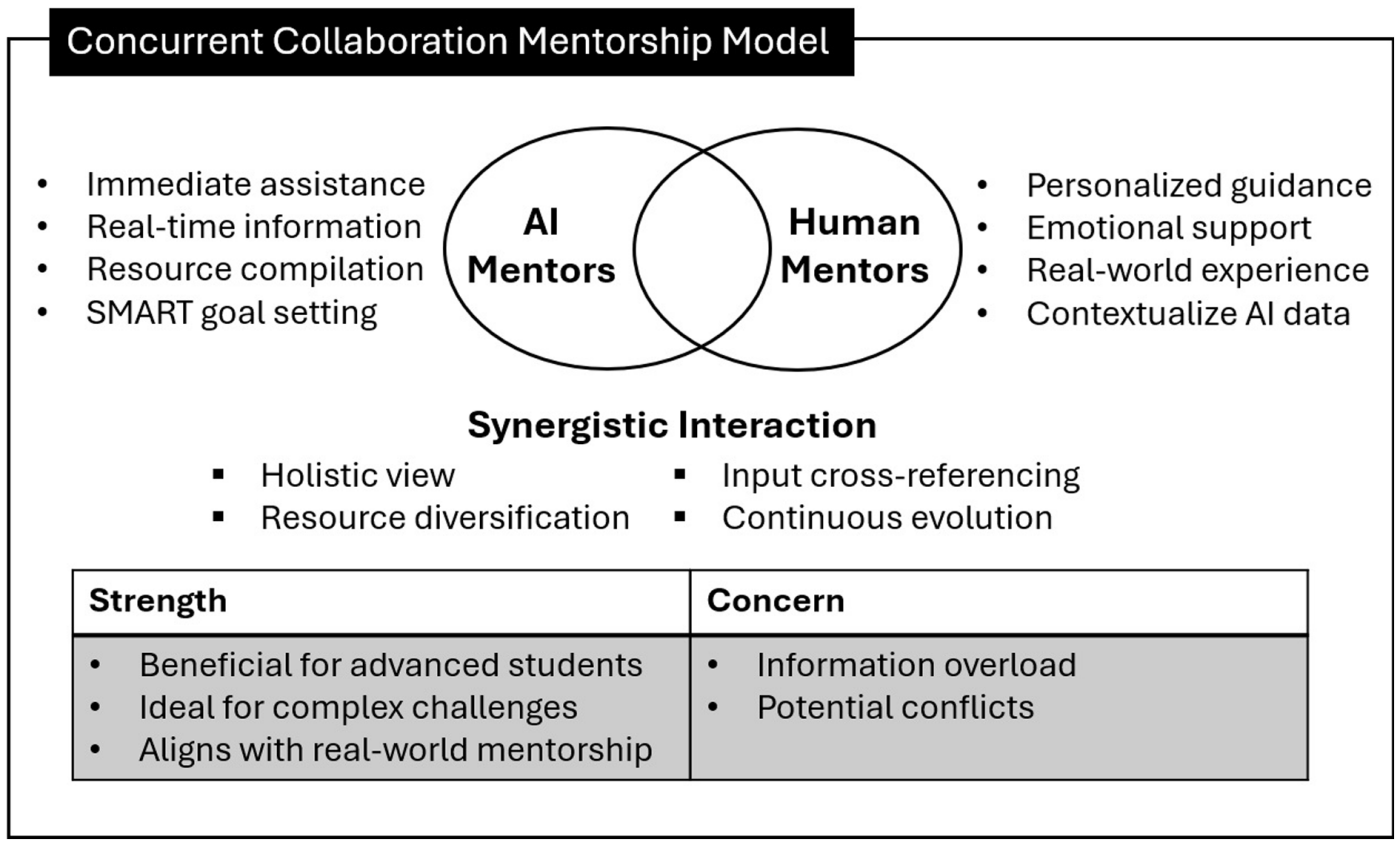
Concurrent Collaboration Mentorship Model showing human-AI collaborative mentorship flow.

The Concurrent Collaboration Mentorship Model (Figure 4), on the other hand, features continuous and simultaneous collaboration between human and AI mentors throughout the entire mentorship process. This model mirrors real-world settings where students often work with multiple mentors, each providing different types of instrumental and psychological support (Eby et al., 2013; Saw et al., 2022). AI offers immediate assistance, compiling resources, crafting structured plans, setting SMART goals, and stimulating creative thinking for career and mentorship development. Human mentors provide personalized and emotional support, utilizing their real-world experience to address complex personal or professional challenges, ensuring the mentorship is empathetic and practical. They also contextualize AI guidance, interpreting and adjusting AI-generated recommendations to fit the unique contexts of each mentee. By leveraging both AI and human insights, the mentorship experience continuously evolves and adapts to meet the mentees’ needs effectively. The dynamic integration of efforts allows for real-time support, diverse resources, and perspectives, fostering holistic development through human-AI interaction. This model is particularly effective for students with a basic understanding of their field, such as second-year students and beyond, and for complex challenges requiring real-time support and diverse resources. However, there is a risk of information overload for mentees and potential conflicts between the guidance provided by human and AI mentors.

These models provide a structured approach to leveraging both human and AI strengths, offering new perspectives on blended mentorship practices for developing personalized IDPs. Our findings highlight the potential of these models to make mentorship more scalable and accessible, particularly in contexts where human mentors are in short supply or where underrepresented minorities require career development support without sufficient resources. These models underscore the importance of this AI integration with mentorship to enhance the developmental process for mentees, ensuring that mentorship is both comprehensive and adaptive to individual needs.

### 4.4 Unique challenges faced by international STEM students

The current myIDP platform’s U.S.-centric design inadequately serves the unique needs of international STEM graduate students, whose career paths are often obstructed by language barriers, cultural differences, social isolation, and restrictive visa conditions (American Council on Education, 2021; Lee, 2020; Rodriguez et al., 2024). Such challenges not only hinder their personal and career development but also potentially weaken the broader U.S. innovation landscape. Given the vital role that international STEM talent plays in driving a robust U.S. economy (National Science Board, 2022), there is an immediate need to reevaluate the myIDP framework to be more inclusive, culturally relevant, and globally responsive, especially by integrating AI support.

The study uncovered significant insights from international student participants. First, the AI responses are often US-centric unless the country is explicitly specified in the questions. This issue persists even when using VPN services to access AI outside of the U.S. Second, the accuracy of career information provided by AI mentors can be problematic, as some information may be incorrect for countries outside the US or for countries sharing similar languages. Third, cultural sensitivity is a critical issue. The advice from AI mentors sometimes fails to consider cultural differences. Addressing these issues could enhance the effectiveness of AI mentors and better support the diverse needs of international STEM students, thereby improving their career outcomes and contributing to the broader academic and professional community.

### 4.5 Limitations and future directions

There are several limitations to our study. First, AI tools are evolving very rapidly. Although we identified several concerns about AI mentors, these concerns are based on findings during the period when participants tested the AI prompts (January 2024–May 2024). These concerns might be addressed in future advancements. Second, prompt engineering is a significant area of research focused on optimizing AI responses. The suggested AI prompts used in this study might need to be updated due to the ongoing evolution of AI tools. Since prompt engineering was not our focus, we provided only basic guidance to student participants, allowing them to freely interact with AI mentors. This approach enabled us to gather diverse insights from their interaction experiences. Future research could explore how prompt engineering can enhance the interaction process with AI mentors.

Third, to promote educational equity, we avoided using paid versions of AI tools that could pose a barrier for minority students. Thus, our study utilized the free AI tool, Gemini, to test its potential in the STEM career development process. Our findings are specific to Google Gemini. Other paid AI tools (such as GPT-4), which also have internet access, may be worth examining in future studies. Fourth, our study focuses solely on the mentee’s perspective. Future studies could focus on the perspectives of mentors. Lastly, our study proposed two conceptual human-AI mentoring models based on student feedback. Further research is needed to evaluate the effectiveness of these models in real-world settings. Potential areas for improvement include enhancing AI personalization capabilities and investigating the impact of human-AI interactions on mentor-mentee relationships. Such research could provide valuable insights into optimizing these models to better support the diverse needs of students and improve overall mentoring outcomes in developing IDPs.

## 5 Conclusion

**Figure 5:**
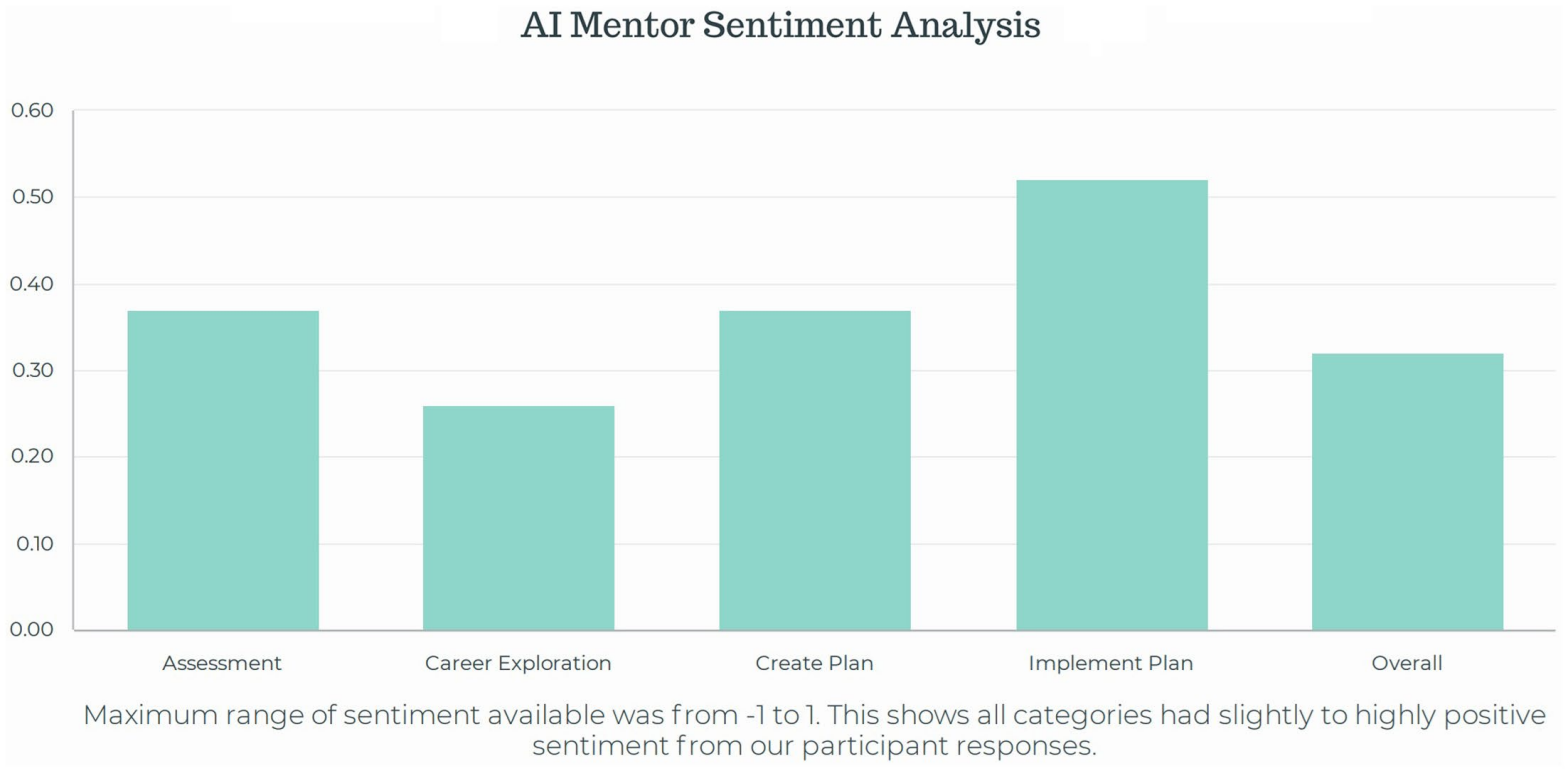
Sentiment scores for AI prompts across myIDP components.

This study makes several theoretical, methodological, and practical contributions to the literature on IDPs, career development, and mentoring in higher education. Theoretically, it proposes integrating AI technology into career planning and higher education mentoring, moving beyond traditional human-centered theories. Two conceptual human-AI mentoring models, the Sequential Integration Mentorship Model and the Concurrent Collaboration Mentorship Model, are introduced. Methodologically, our study develops an AI-integrated myIDP framework by incorporating prompt submissions to Google Gemini. Feedback from student participants highlighted its strengths and limitations through thematic analysis, while sentiment analysis assessed the usefulness of each AI prompt. Practically, this study demonstrates the promising approach of using Google Gemini to optimize IDP practice (Figure 5), providing immediate feedback and information to empower students in their career development and transcend the limitations of human mentors. The proposed hybrid human-AI mentoring models show potential in supporting more underrepresented minority students in their STEM career development process, promoting broader participation in STEM fields. These models could be further examined in real-world settings in the future.

Despite the strengths discussed, users must be aware of concerns regarding accuracy, bias, privacy, equity, and algorithmic influence. To enhance this process, we encourage graduate students to reassess their SMART goals and action plans with their human mentors for personalized support. The sentiment analysis shows that there are clear areas of effectiveness in using AI for mentorship, though further research must explore ways to improve AI effectiveness. While AI technology can benefit the career planning process within IDPs, human mentors remain vital for providing comprehensive support during plan implementation, encompassing both instrumental and psychosocial aspects. Therefore, AI technology should supplement, not replace, the essential role of human mentors in the mentoring process. Future research may also investigate the optimal balance between AI and human mentorship to bolster career development experiences.

## Data Availability

The raw data supporting the conclusions of this article will be made available by the authors, without undue reservation.

## Footnotes

^1^ https://myidp.sciencecareers.org/
